# Molecular Evolutionary Routes That Lead to Innovations

**DOI:** 10.1155/2012/483176

**Published:** 2012-10-04

**Authors:** Frédéric Brunet, Hideki Innan, Ben-Yang Liao, Wen Wang

**Affiliations:** ^1^Institut de Génomique Fonctionnelle de Lyon, Ecole Normale Supérieure de Lyon, 32-34 Avenue Tony Garnier, 69007 Lyon, France; ^2^Hayama Center for Advanced Studies, The Graduate University for Advanced Studies, Kanagawa 240-0193, Japan; ^3^Division of Biostatistics and Bioinformatics, Institute of Population Health Sciences, National Health Research Institutes, 35, Keyan Road, Zhunan, Miaoli County 350, Taiwan; ^4^Max-Planck Junior Scientist Group on Evolutionary Genomics, Kunming Institute of Zoology, Chinese Academy of Sciences, Yunnan, Kunming 650223, China

In biology, it is always fascinating to observe the continuous changes occurring at different levels in living organisms. In demonstration of this interest, one question we regularly ask is the following:

*“What are the events that contribute to the evolution of new functions and adaptive evolutionary innovations?”*

Deep down at the molecular level, we are referring to the various genetic and molecular mechanisms that occasionally either duplicate preexisting genes, or lead to the origination of new sequences. Altogether, these new and duplicated genes represent a substantial fraction of every genome sequenced. They are of multiple origins ranging from whole-genome duplications, which are documented in many eukaryotes, to various other modes of duplication—mostly single full or partial gene duplications—by DNA-based or retroposition events.

Each month, new and exciting articles dealing with these topics are published in different journals, and it has now been several years since a special issue collecting this research into a single volume has been published. Our belief is that it is now the time for the publication of a new special issue that would bring together researchers working in this field, and provide up-to-date research on the subject. For this special issue, we allowed these authors the liberty to offer either review papers or pure research articles. Others were also encouraged to use a rather unconventional format, bringing together a review of their published work, but updated with new insights into their latest discoveries. Experts in the field performed in-depth reviews of these submissions to ensure the articles would be of the highest quality. Among the 18 articles that went through this reviewing process, 17 were accepted. We feel that this represents the high quality of the work offered to us by the invited authors. 

Above all, we are immensely grateful to all authors and reviewers who contributed to this special issue. We would like to take the chance to express our sincerest gratitude to each and every person involved in this project. The effort they have put forth is an expression of the dedicated passion we all share for this field of research. Additionally, we deeply hope that everyone will enjoy reading each and every one of these excellent articles as much as we did.

D. Kordiš and J. Kokosar examined the intron dynamics in the domesticated genes of eutherians, showing some gained with positional bias.

K. S. Swithers et al. reviewed the functional role of reticulate evolution, focusing on horizontal gene transfers, in creating novelties and complexities.

R. Ponce et al. reviewed novel genes, mostly in *Drosophila* and primates, providing details of the chimeric gene *Sdic*.

L. Chen et al. reviewed and discussed the important role alternative splicing may have in generating transcriptome and organism complexity during eukaryotic evolution.

R. Dubruille et al. reviewed and presented their latest results on the evolution of the hiphop/K81 telomere capping genes in *Drosophila*.

L. Huminiecki and G. C. Conant demonstrated the rise to complex innovations in cellular networks that can only be evolved from whole-genome duplications using examples in fungi and vertebrates.

G. V. Markov and R. J. Sommer reviewed the evolution of novelties among conserved gene families in insects and nematodes genomes.

P. C. Sharma et al. provided a nice example of the potential evolution of microsatellites in duplicated regions of the rice genome.

E. Dubois et al. discussed the impact the domestication of the transposase of a piggyBac had on the spread of Tc1/mariner elements throughout the germline of a Paramecium.

G. Diss et al. provided some evidence that posttranslational regulatory control on a function might influence the divergence between paralogous genes.

O. M. Ramos and D. Ferrier gave an overview on the terminology, mechanisms, and relative frequencies of gene duplications at different scales in shaping animal genome architecture.

S. Good et al. gave force details of the complex evolution of the relationship of the relaxin family genes and their receptors after the fish-specific whole-genome duplication.

E. Betrán et al. considered the evolution of Y chromosome palindromes giving emphasis on the evolutionary role of gene conversion.

M. P. Francino presented very nicely the novelties brought by duplications and horizontal gene transfers and how this can impact a bacterial community.

S. Oulion et al. reconsidered the complex scenarios of duplications and loss events of the FGF family genes during the evolution of the Metazoa.

V. Katju brought a multidimensional consideration of the gene duplication process.

D. Chalopin et al. provided nice examples of genetic innovation in vertebrates by domestication of transposable elements.

*Frédéric Brunet* *Frédéric Brunet* *Hideki Innan* *Hideki Innan* *Ben-Yang Liao* *Ben-Yang Liao* *Wen Wang* *Wen Wang*

